# Large cystic-based gastrointestinal stromal tumor: A case report

**DOI:** 10.3892/ol.2014.1798

**Published:** 2014-01-15

**Authors:** XINQI CHEN, HUIZENG LV, WENHAI ZHANG, JIE CAO

**Affiliations:** 1 Department of Digestive Surgery, The Fifth Affiliated Hospital of Guangzhou Medical University, Guangzhou, Guangdong 510700, P.R. China; 2 Department of Gastrointestinal Surgery, Affiliated Guangzhou First People’s Hospital, Guangzhou Medical University, Guangzhou, Guangdong 510180, P.R. China

**Keywords:** gastrointestinal stromal tumor, exophytic tumor, extensive cystic change, c-kit

## Abstract

A 60-year-old male with increasing abdominal distension was admitted to The Fifth Affiliated Hospital of Guangzhou Medical University (Guangzhou, China). The abdominal sonogram exhibited a huge abdominal cystic-based mass with solid components in the left upper quadrant. A contrast-enhanced computed tomography scan of the abdomen revealed a large heterogeneous cystic solid tumor, but the source of the tumor could not be determined. The laparotomy demonstrated a huge cystic-based tumor with an integrated cystic wall, arising from the posterior wall of the gastric body. Multiple septa and ~3,500 ml of yellowish fluid were found in the cystic cavity. Pathological analysis showed that the tumor contained a mixture of polygonal and spindle cells. Immunohistochemical study indicated that the tumor cells were positive for CD117 and CD34. The final diagnosis was gastric gastrointestinal stromal tumor. The patient recovered well and no recurrence or metastasis was identified following a 12-month follow-up.

## Introduction

Gastrointestinal stromal tumors (GISTs) are the most common type of mesenchymal tumors of the gastrointestinal (GI) tract. Different from other mesenchymal tumors, such as GI smooth muscle and nerve sheath tumors, GISTs are a spontaneous differentiation of stromal tumors (1). GISTs may appear throughout the GI tract, between the esophagus and the rectum, but are most frequently found in the stomach (50%) (2). The majority of GISTs are centered in the submucosa or the muscularis propria and appear as nodular or lobulated solid masses (3). Cystic-based tumors are uncommon in GISTs, however, the current study presents a case of large gastric GIST with exophytic growth and extensive cystic change.

## Case report

A 60-year-old male, who exhibited increasing abdominal distension for one month, with a huge congenital right inguinal hernia, was admitted to The Fifth Affiliated Hospital of Guangzhou Medical University (Guangzhou, China). The patient denied any abdominal pain, vomiting or constipation. Physical examination revealed a large firm mass that occupied almost the entire abdomen; the mass extended from the bottom of the xiphoid process to 2.5 cm over the pubic symphysis. In addition, part of the bowel was apparent in the right side of the scrotum. Laboratory results indicated that the patient was anemic (hemoglobin, 95 g/l), with an increased peripheral blood platelet count (420×10^9^/l) and elevated blood (250 U/l) and urinary (1,325U/l) amylase levels. The serum levels of specific tumor markers (CEA, AFP and CA19-9) were within the normal range. However, the level of CA125 (142.3 U/ml) was significantly higher. The patient provided written informed consent.

**Figure 1:**
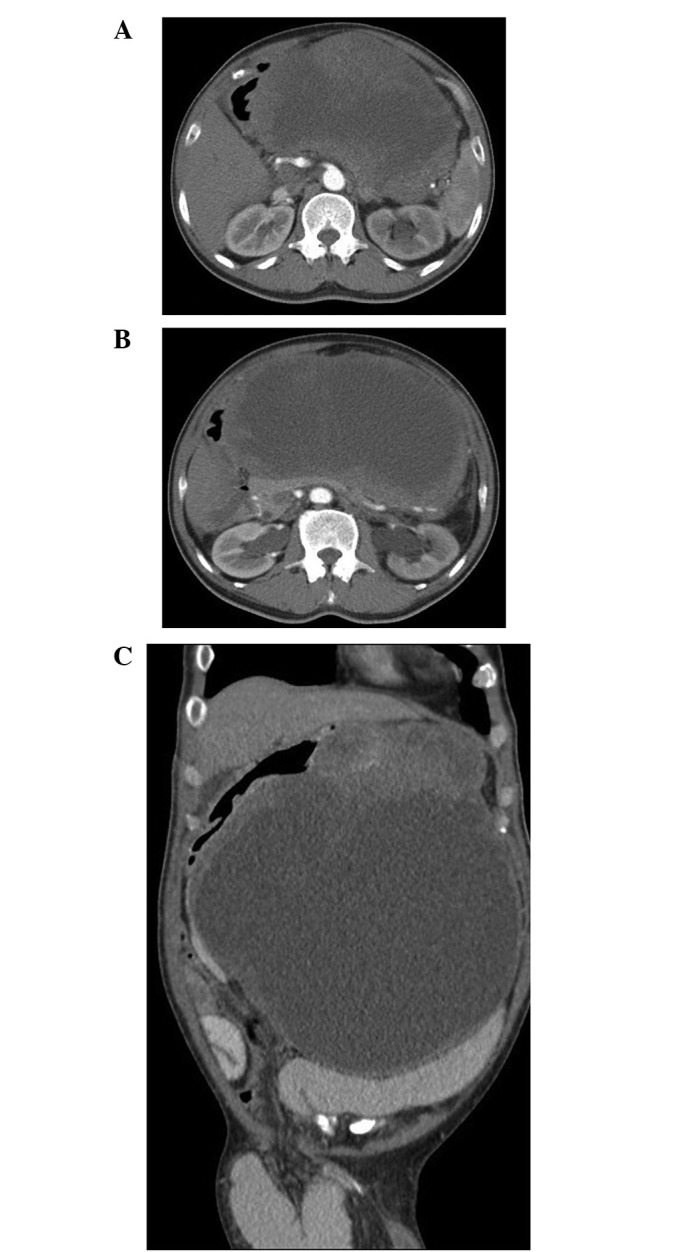
Computed tomography scan shows a large cystic-based tumor with a wall of uneven thickness. The tumor, with an unclear boundary between the stomach and spleen, (A) compressed the stomach to the right upper quadrant, leading to gastric lumen narrowing, (B) extended backward and compressed the pancreas and (C) almost occupied the entire abdominal cavity.

**Figure 2:**
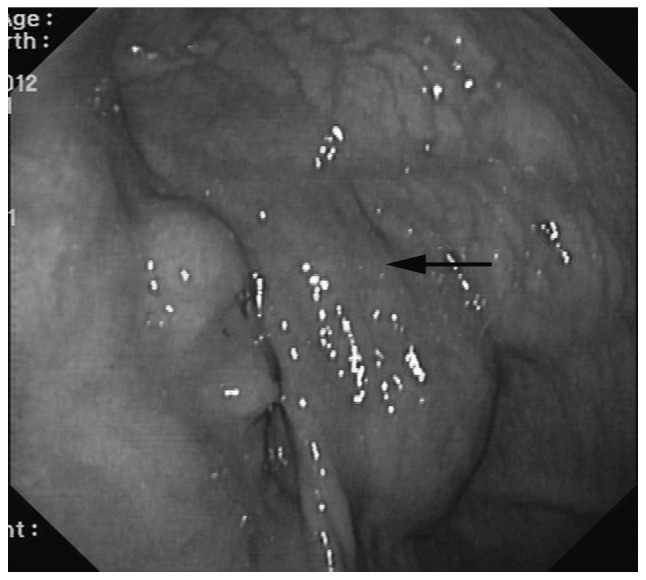
Endoscopy shows local eminence of the gastric mucous membrane and reveals superficial veins, indicated by the black arrow.

The abdominal sonogram showed a huge abdominal cystic-based mass with solid components in the left upper quadrant. The cystic portion was irregular in shape with a wall of uneven thickness and an unsmooth inner surface. The size of the solid portion was ~8.3×4.6×9.0 cm^3^, close to the left lobe of the liver. A contrast-enhanced computed tomography (CT) scan of the abdomen revealed a 28.5×22.8×19.2-cm^3^ heterogeneous cystic solid tumor. Partial septum was found in the cyst cavity. The mass, with an unclear boundary between the stomach and spleen, compressed the left lobe of the liver and the stomach, pancreas and kidneys, leading to narrowing of the gastric lumen and bilateral hydronephrosis. No evidence of swelling of the regional lymph nodes or involvement of the major vessels was identified (Fig. 1). An endoscopy suggested compressed gastric wall, eminence of gastric mucous membrane and superficial veins (Fig. 2). According to these examinations, the tumor was difficult to diagnose qualitatively and its primary organ was difficult to determine.

**Figure 3:**
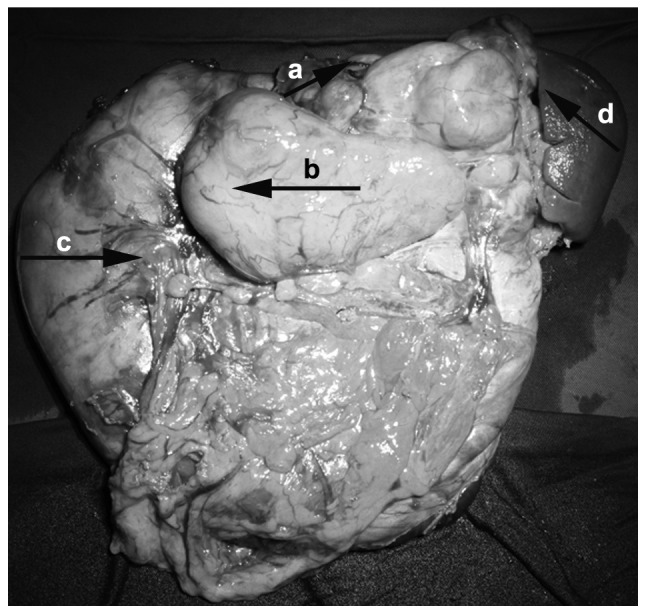
Resected specimen indicated as follows: a, section of the stomach wall; b, solid portion of the tumor; c, cystic portion of the tumor; and d, the spleen.

**Figure 4:**
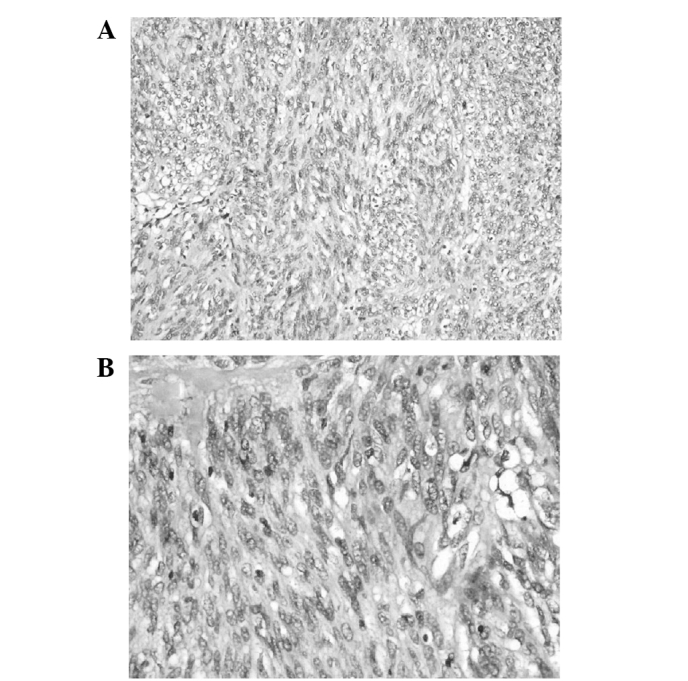
(A) Pathological analysis demonstrated a gastrointestinal stromal tumor with a mixture of polygonal and spindle cells (hematoxylin and eosin; magnification, ×200). (B) The cells appeared atypical (hematoxylin and eosin; magnification, ×400).

The patient underwent an exploratory laparotomy with L-shaped incision in the left upper quadrant. The laparotomy revealed a huge cystic-based tumor with an integrated cystic wall, arising from the posterior wall of the gastric body. Multiple septa and ~3,500 ml of yellowish fluid were found in the cystic cavity. No clear boundary was identified between the tumor and splenic hilum. The patient’s tumor and spleen were completely resected. The resected tumor weighed 122 g and exhibited a fish-flesh cut surface (Fig. 3). Pathological analysis demonstrated a highly malignant GIST, containing a mixture of polygonal and spindle cells. The cells appeared atypical and a certain amount of nuclear division was observed (Fig. 4). The spleen was not infiltrated. Immunohistochemical (IHC) study showed that the tumor cells were positive for CD117, CD34, DOG-1, vimentin and S-100, while negative for desmin, smooth muscle actin and CK. The patient recovered well and underwent a tension-free inguinal hernia repair one month later.

## Discussion

GISTs are the most common type of mesenchymal tumors. Without a myogenic or neurogenic nature, GISTs are considered to originate from interstitial Cajal cells of the wall of the GI tract (4). Microscopically, the majority of GISTs have a relatively uniform appearance, exhibiting a spindle cell type (2,5). At present, mutations of the c-KIT/KIT proto-oncogene and expression of the c-kit protein (CD117) are regarded as ubiquitous features of GISTs (6,7). However, patients with GISTs often exhibit non-specific features clinically and in laboratory tests.

The clinical manifestation of GISTs, such as abdominal distension, lower abdominal pain, GI bleeding and abdominal mass, varies with the size and location of tumors (8). The median tumor size presented in symptomatic patients is 5 cm (6). The patient of the current study presented to our hospital for treatment with a tumor ~28 cm in size, with abdominal distension, belching, anorexia and anemia, which were mainly caused by the compression of the tumor. In addition, the patient was with congenital inguinal hernia. The exophytic tumor had grown in the abdominal cavity, causing the small intestine to be squeezed into the scrotum, making sufficient space for the growth.

Due to the wide range of symptoms and its rarity, the diagnosis of GISTs require a high degree of suspicion. The primary mode of diagnosis and assessment of the severity of the disease is by contrast-enhanced CT scan of the abdomen and pelvis (6,9). In the present case, CT scan revealed a large heterogeneous cystic solid tumor, but the source of the tumor could not be determined. Prior to the surgery, the tumor was suspected as a pancreatic pseudocyst. The accurate diagnosis of GIST must be based on tumor morphology and immunohistochemistry (CD117 and/or DOG1) (10,11). The present case was finally diagnosed as GIST based on the results of IHC staining.

To date, complete surgical resection is the most effective treatment for GISTs and has a major impact on the prognosis of patients and tumor recurrence (12). In the current report, due to the adhesion between the tumor and splenic vascular, combined organ resection was performed to ensure the complete excision of the tumor. According to the risk classifications of GISTs (11), the patient’s case was high-risk. The results from several previous studies suggest that adjuvant imatinib is useful in specific high-risk patients following surgical resection (13,14). However, other previous studies have confirmed that the biological behavior of cystic GIST is indolent with a low risk of malignancy and that surgical resection may achieve a favorable prognosis (15). The present patient was not administered imatinib following the surgery. Currently, the patient has been followed-up for one year and no evidence of tumor recurrence or metastasis has been found.

In conclusion, large cystic-based GISTs are rare and GISTs must be considered as one possibility when cystic tumors of unknown origin are identified in the abdomen.
